# Revision surgery after pregnancy in a patient with congenital kyphoscoliosis: A case report

**DOI:** 10.1097/MD.0000000000005624

**Published:** 2016-12-09

**Authors:** Zhikun Li, Fei Wang, Wei Xu, Yifan Li, Xiaodong Zhu

**Affiliations:** a Department of Orthopaedics, Tongren Hospital, Shanghai Jiao Tong University School of Medicine; b Department of Orthopaedics, Changhai Hospital, Second Military Medical University, Shanghai, People's Republic of China.

**Keywords:** congenital scoliosis, pregnancy, revision surgery, rod breakage

## Abstract

**Rationale:**

Rod breakage during pregnancy and delivery has never been described in a patient who has undergone surgery for congenital scoliosis (CS). Here, we present an unusual but significant case of revision surgery.

**Patient concerns:**

A 29-year-old woman presented with low back pain during pregnancy after posterior osteotomy, correction and fusion at T9 to L5 for CS. Radiographs during follow-up, 4 months after the patient gave birth, demonstrated rod breakage.

**Diagnoses:**

Rod breakage after orthopaedic surgery of congenital kyphoscoliosis

**Interventions:**

The patient was taken into the operating room for replacement of the broken rods, recovery of sagittal balance, bone graft fusion, and improvement of stability by cross-connection. The patient recovered fully by the 3-month postoperative follow-up.

**Outcomes:**

In follow-up, the instruments were in good condition, the orthopedic effect was not lost, and low back pain relief was observed.

**Lessons:**

We opine that the rod breakage during pregnancy resulted from weight gain and a lack of an anterior approach to the supportive bone graft. Therefore, female patients with spinal surgery should visit the hospital for advice before pregnancy.

## 1 Introduction

The etiology of congenital scoliosis (CS) is unknown, although a variety of factors have been implicated in the development of vertebral abnormalities.^[1]^ The mainstay of surgical treatment remains early diagnosis before severe curvature and deformity develop. Occasionally, patients present with large deformities that require more significant procedures.^[2]^ Statistics indicate that the female:male ratio is 1.4:1,^[3]^ meaning that more than half of patients are women. Therefore, many patients who are treated with surgery will face pregnancy and delivery.

Previous studies have suggested that pregnancy does not significantly increase fused scoliosis curvatures or the remaining unfused curvatures.^[4,5]^ However, there have been no reports concerning this situation in CS. Here, we present a case of revision surgery in a female patient with congenital kyphoscoliosis with an unusual presentation after pregnancy and delivery.

## 2 Case report

**Figure 1:**
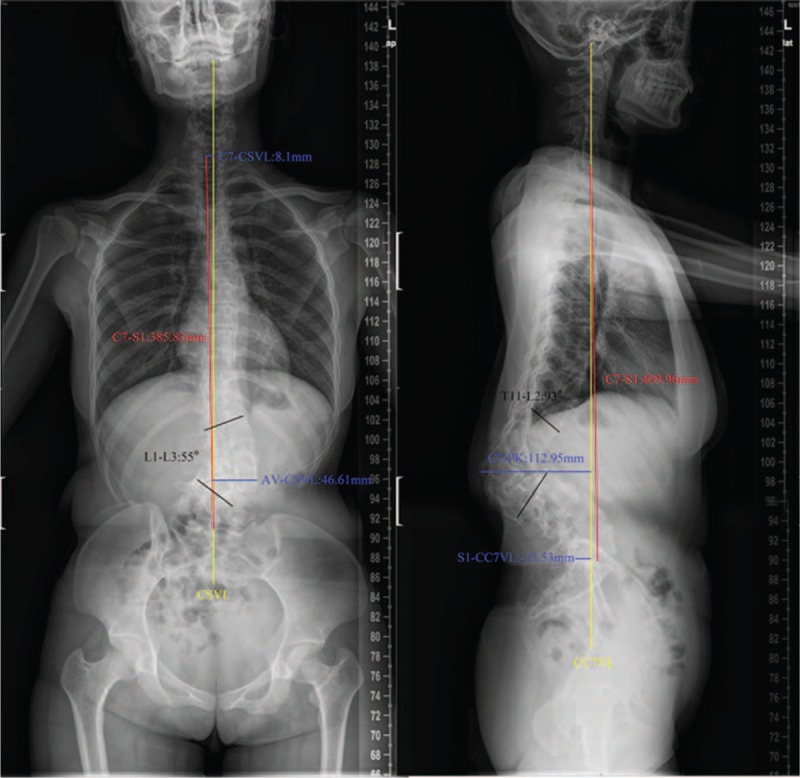
Lateral and anteroposterior X-ray, preoperation, lumbar kyphosis with a Cobb angle of 86° (L1–L2), and lumbar scoliosis with a Cobb angle of 55° (L1–L3).

We present the case of a 29-year-old woman who was diagnosed with congenital kyphoscoliosis in childhood. She remained untreated until the deformity increased and her low back pain worsened sharply. At her first outpatient visit, plain radiographs of the spine showed the following in the coronal plane: lumbar scoliosis with a Cobb angle of 55° (L1–L3), deviation of the apical vertebra to the central sacral vertebral line (CSVL) of 46.61 mm, and location of C7 in the CSVL. In the sagittal plane, lumbar kyphosis with a Cobb angle of 93° (T11–L2), deviation of the most kyphotic vertebra to the C7 vertical line of 112.95 mm, and a distance between the C7 vertical line and the posterior superior margin of the sacrum of −15.53 mm were observed (Fig. 1, Table 1), suggesting a need for surgical correction.

**Table 1:**
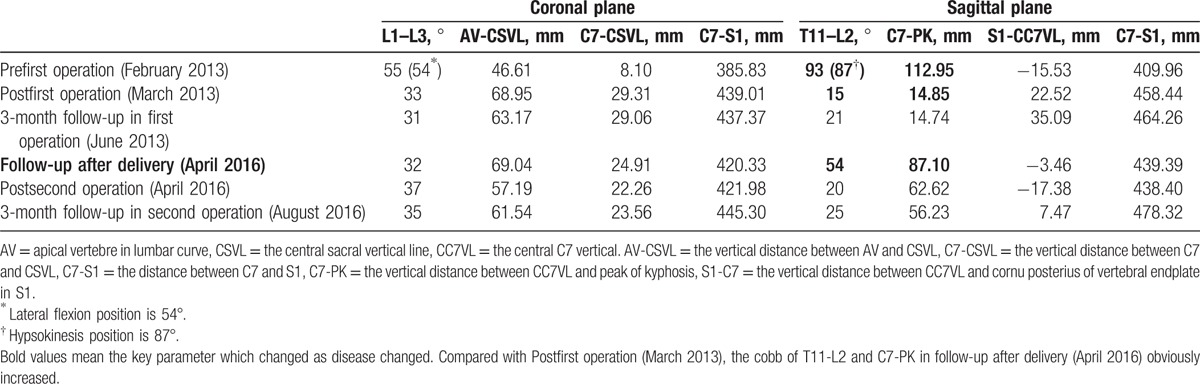
Parameters on coronal and sagittal plane.

After hospitalization of the patient, further examination was performed. On clinical physical examination, the surface lacked abnormal hair and hyperpigmentation, and a pelvic tilt to the left was observed. Left lumbar and severe kyphoses were noted. Shoulder imbalance, stretching and bending of the back with mild activity, and mild tenderness of the lumbar muscle were also observed. Limb muscle tension and myodynamia and feeling in the limbs and saddle area were all normal. No reflection or pathological characteristics were abnormal either.

**Figure 2:**
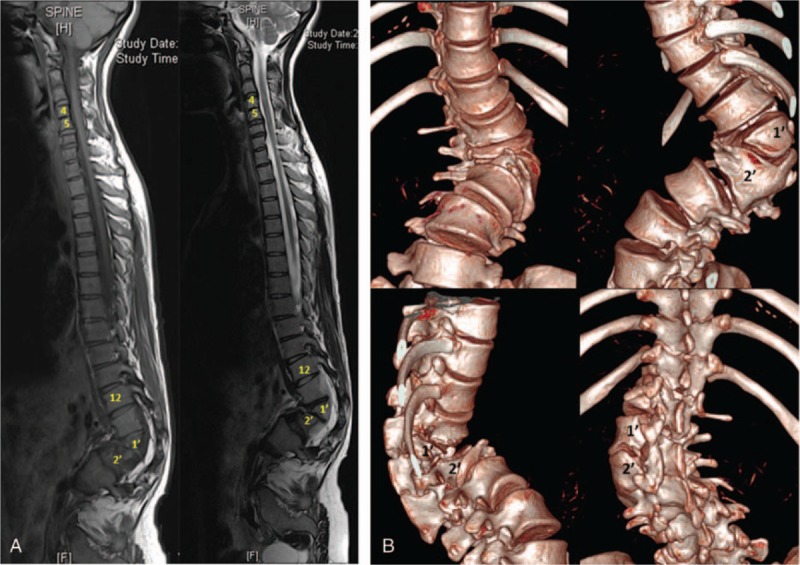
1–2 A thoracolumbar 3-dimensional computed tomography reconstruction and spinal magnetic resonance imaging revealed dysplasia of the first and second lumbar vertebral bodies (hemivertebra) and of the fourth and fifth cervical vertebral bodies (failure of segmentation), in addition to thoracolumbar kyphoscoliosis.

**Figure 3:**
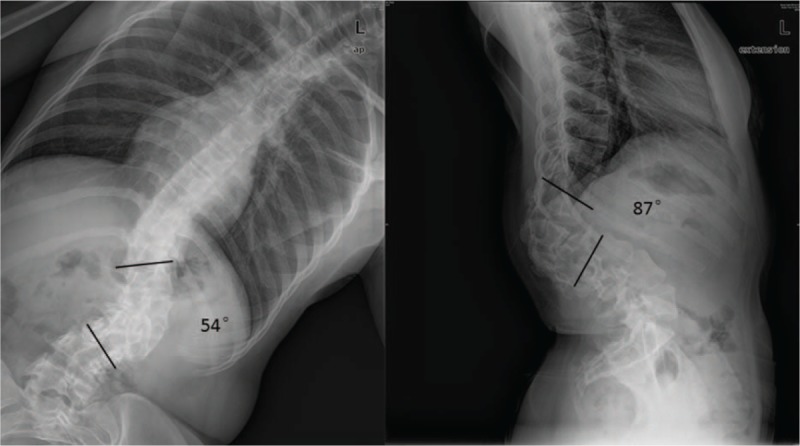
Flexion/extension X-ray.

**Figure 4:**
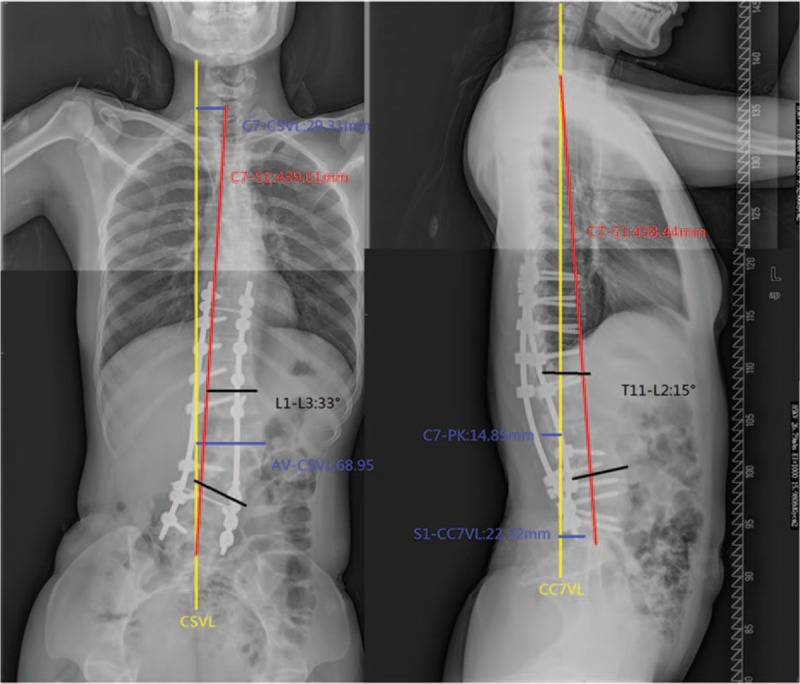
Lateral and anteroposterior X-ray, postoperation.

**Figure 5:**
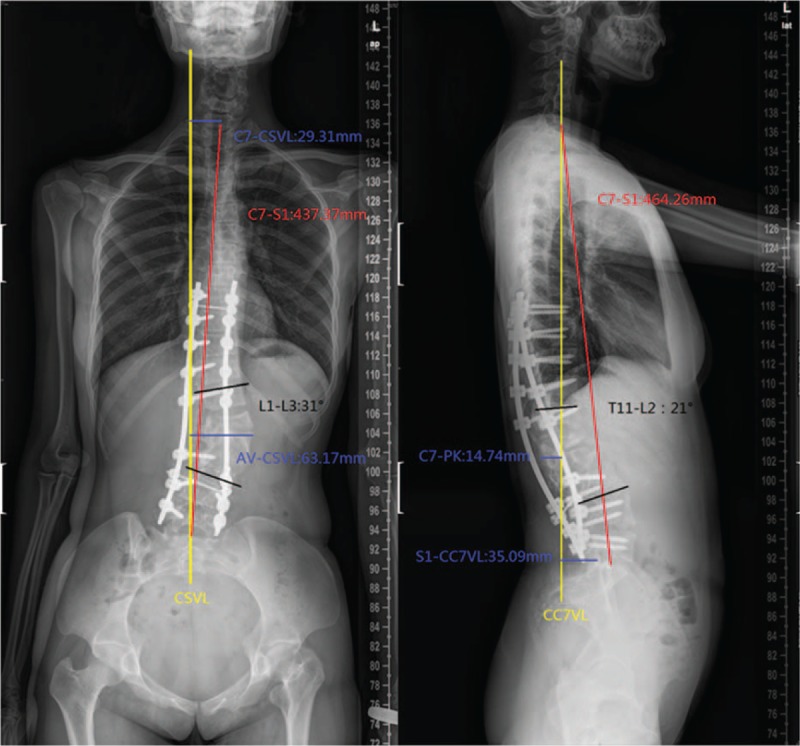
Lateral and anteroposterior X-ray, 3-month postoperative follow-up.

On imaging examination, thoracolumbar 3-dimensional computed tomography reconstruction and spinal magnetic resonance imaging revealed L1 and L2 vertebral body dysplasia (hemivertebra), C4 and C5 vertebral body dysplasia (failure of segmentation), and thoracolumbar kyphoscoliosis (Fig. 2). Flexion/extension X-ray showed that the kyphoscoliosis was rigid (bending plane L1–L3 Cobb angle was 54°; extension plane T11–L2 Cobb angle was 87°) (Fig. 3). On February 27, 2013, posterior osteotomy, correction, and fusion were performed as follows: the bilateral articular process was exposed from T9 to L5. Pedicle screws were implanted in each vertebra (T9, T10, T11, T12, L1, L3, L4, and L5). To resect the L1 and L2 hemivertebra, pedicle subtraction osteotomy (PSO) was used. The cortical bone of the hemivertebra was removed by abrasive drilling, and the cancellous bone of the hemivertebra was drawn out by curetting. Deformity correction was performed. Suitable rods were curved into a normal spinal curve using a plate bender. A cantilever technique was performed during the rod installation. Posterolateral bone graft fusion was performed with both autogenous bone and allograft bone. As the bleeding volume was approximately 700 mL, transfusion of 800 mL red blood cells, 400 mL plasma, and 230 mL autoblood was conducted. Spinal cord monitoring was performed during surgery (Fig. 3) using the Expedium spinal system (Johnson & Johnson, New Brunswick, NJ). Postoperative plain radiographs of the spine showed the following in the coronal plane: lumbar scoliosis with a Cobb angle of 33° (L1–L3), deviation of the apical vertebra to the CSVL of 68.95 mm, and a distance between C7 and the CSVL of 29.31 mm. In the sagittal plane, lumbar kyphosis with a Cobb angle of 15° (T11–L2), deviation of the most kyphotic vertebra to the C7 vertical line of 14.85 mm, and a distance between the C7 vertical line and the posterior–superior margin of the sacrum of 22.52 mm were observed (Fig. 4, Table 1). The instruments were in good condition at the 3-month postoperative follow-up (Fig. 5). Relative parameters are shown in Table 1.

**Figure 6:**
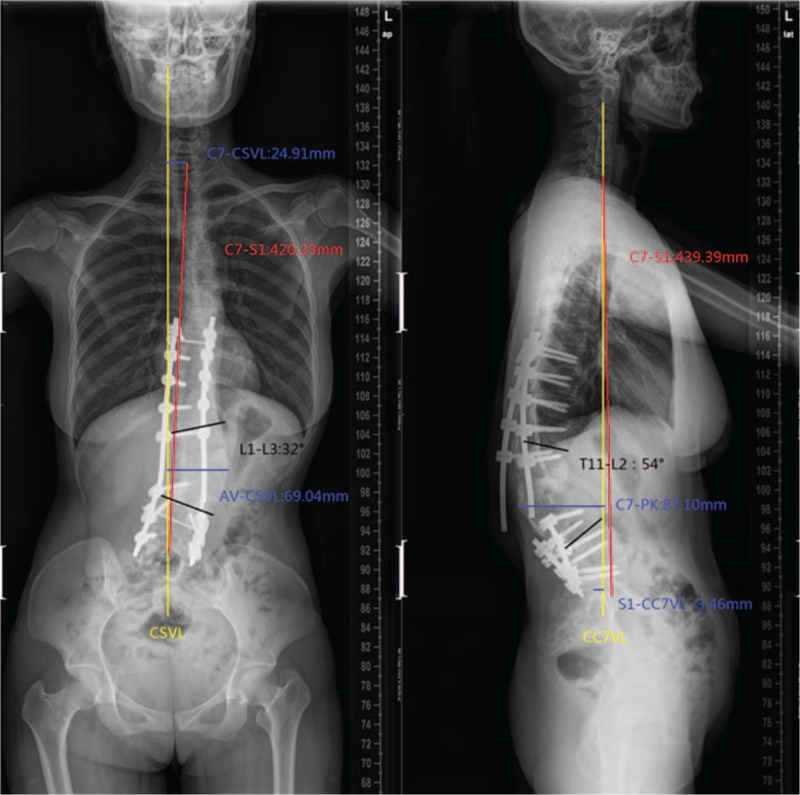
Lateral and anteroposterior X-ray, the rods were broken, kyphoscoliosis had increased.

**Figure 7:**
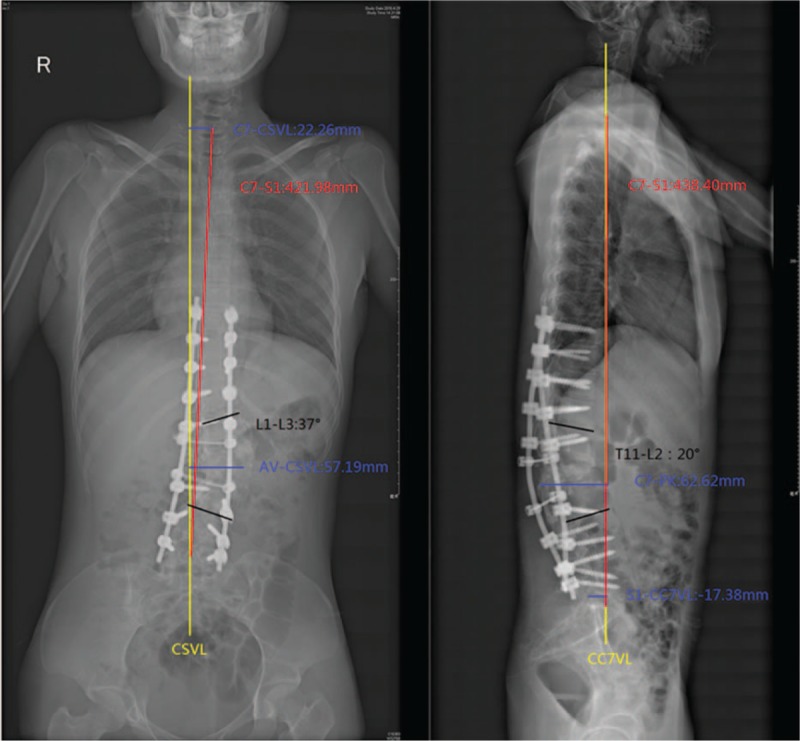
Lateral and anteroposterior X-ray, postrevision surgery.

**Figure 8:**
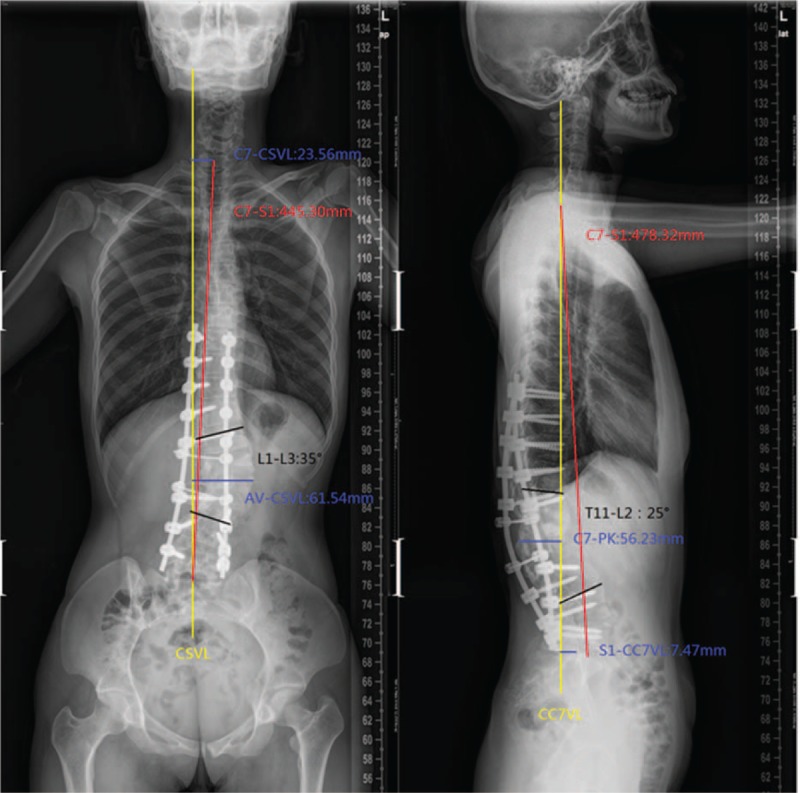
Lateral and anteroposterior X-ray, 3-month postrevision surgery follow-up.

In February 2015, due to increasingly severe back pain (beginning in the fourth month), the patient had to go to the hospital during pregnancy. To avoid radiation, the patient refused to undergo plain radiographs. We suggested that she should undergo reexamination after delivery. In April 2016 (4 months after delivery: eutocia, no anesthesia, male baby, 2.9, and 62.5 kg before delivery), plain radiographs of the spine showed rod breakage, increased kyphoscoliosis, and spinal instability at follow-up (Fig. 6). Her plain radiographs of the spine also showed the following in the coronal plane: lumbar scoliosis with a Cobb angle of 32° (L1–L3), deviation of the apical vertebra to the CSVL of 69.04 mm, and a distance between C7 and the CSVL of 24.91 mm. In the sagittal plane, lumbar kyphosis with a Cobb angle of 54° (T11–L2), deviation of the most kyphotic vertebra to the C7 vertical line of 87.1 mm, and a distance between the C7 vertical line and the posterior–superior margin of the sacrum of −3.46 mm were observed, suggesting a need for revision surgery. On April 25, 2016, a posterior revision was performed. First, the broken rods were removed after exposure. Second, suitable rods were reinstalled, and sagittal balance was recovered. Third, the reprocessed right partial ilium and artificial bone were mixed to perform bone graft fusion on the back side. Lastly, cross-connection was used to improve the stability between L1 and L2. Spinal cord monitoring was performed during surgery. The bleeding volume was approximately 300 mL. Plain radiographs of the spine showed the following in the coronal plane: lumbar scoliosis with a Cobb angle of 37° (L1–L3), deviation of the apical vertebra to the CSVL of 57.19 mm, and a distance between C7 and the CSVL of 22.26 mm. In the sagittal plane, lumbar kyphosis with a Cobb angle of 20° (T11–L2), deviation of the most kyphotic vertebra to the C7 vertical line of 62.62 mm, and a distance between the C7 vertical line and the posterior–superior margin of the sacrum of −17.38 mm were observed using the Expedium spinal system (Johnson & Johnson) (Fig. 7). At the 3-month postoperative follow-up, the instruments were in good condition, the orthopedic effect was not lost, and low back pain relief was observed. Relative parameters are listed in Table 1 (Fig. 8).

## 3 Discussion

Vertebral anomalies causing CS are classified on the basis of failures of formation, segmentation, or both. The natural history depends on the type of anomaly and the location of the anomaly.^[6]^ Congenital vertebral anomalies have the potential to progress, so careful assessment and monitoring are essential, and early intervention may be desirable.^[7]^ The mainstay of treatment is either observation or, in the case of curve progression (>10°/y), surgery.^[8]^ The hallmark of surgical treatment is early intervention before the development of large curvatures. The surgical options for congenital spine deformities are numerous and depend on the type of anomaly, the degree of deformity, and the age of the patient. Posterior hemivertebra resection, correction, and fusion are together the mainstream method of treatment for adult patients.^[2,9]^

Given that congenital vertebral deformity has the characteristic of progressing quickly, the best treatment time is before puberty. Therefore, most female patients undergo orthopedic surgery before adulthood. These female patients may think about the following questions: Will orthopedic surgery affect pregnancy? Will pregnancy aggravate the curvature?

Danielsson and Nachemson used a contrast method for 136 surgically treated women and 111 brace-treated women. The results showed that there was no correlation between progression of the major or lumbar curve and the number of pregnancies or between curve progression and the age at first pregnancy.^[5]^ In addition, Betz et al reported that the age of the patient at the time of the first pregnancy did not influence the risk of progression and that the stability of the curve before pregnancy did not decrease the risk of its progression during pregnancy. In patients who had undergone spinal fusion, progression in the unfused portion of the spine was negligible.^[10]^ Lebel et al^[11]^ suggested that scoliosis is not a risk factor for adverse pregnancy outcomes, and specifically labor dystocia.

However, the case described here showed an exception—rod breakage. The patient had symptoms of low back pain in the fourth month of pregnancy, 2 years postoperation. In particular, dual-side connecting rods broke at the level of the osteotomy (L1/L2) after delivery. The Cobb angle of T11 to L2 in the sagittal plane increased to 54° (it was 15° after the first surgery), and the distance between the kyphosis and the C7 vertical line increased to 87.10 mm (it was 14.85 mm after the first surgery); these findings demonstrate serious loss of balance and serious vertebral instability in the sagittal plane. What was the cause of the rod breakage? An average of 30° to 40° correction can initially be achieved with 1-level PSO^[12–14]^; as the current case involved sagittal lumbar kyphosis with a Cobb angle of 55°, the operative plan of L1/L2 use of PSO was appropriate.^[2]^ On the other side, the kyphoscoliosis dramatically improved after operation (preoperation T11–L2 angle of 93°, postoperation T11–L2 angle of 15°). The correction was satisfactory and was not lost by the 3-month postoperative follow-up. Moreover, there was no back pain after the operation. Adding-on could be ruled out because the patients’ bones were fully mature.^[15]^ In our opinion, the weight gain of pregnancy was the most likely reason for the rod breakage. Generally, data show an average gestational weight gain of 19.7 ± 5.1 kg.^[16]^ Here, the patient's weight was 46 kg before pregnancy, and her weight was 62.5 kg before delivery, indicating a 16.5-kg increase. Abdominal weight gain could have increased the stress concentration and overtaxed the instruments, resulting in kyphosis progression and, ultimately, rod breakage. At last, due to the different fixation strategies between idiopathic and CS, with resection of a deformed vertebra in CS, the osteotomy zone cannot be implanted with pedicle screws, and stress concentration will appear in this zone. Does pregnancy increase the incidence of adverse events postoperatively? There is a lack of literature on this subject. Can a patient avoid rod breakage if an anterior approach is added to support the bone graft in the first operation? We are confident that we can reduce the risk if we add this feature. This is regrettable, however. In recent years, with the improvement of medical technology, more and more female scoliosis patients have undergone treatment with surgery. The abovementioned idiopathic scoliosis was not a risk factor for delivery in a previous study, and the curve will not progress in pregnancy. However, the present study's subject was CS, and research on the relationship between CS and delivery is lacking. To a certain extent, this article reflects a preliminary study. Our research specifically suggests that for women with CS, connecting rods can easily break in the osteotomy zone during pregnancy and delivery if fusion is not sufficient in the osteotomy zone. For female patients with congenital spinal deformity who wish to get pregnant, we suggest attempting sufficient fusion in the osteotomy zone, such as via anterior fixation support and reinforcement with cross-connection.

Spine X-rays should be taken before pregnancy and after delivery. Measurement of the sagittal and coronal parameters preoperation can help to evaluate balance and the fusion situation in the osteotomy zone. Postoperative images can specifically be compared with preoperative images. Whether controlling one's weight during pregnancy can reduce the occurrence of adverse events has not been studied, but we speculate that weight control is a method that can reduce the stress in the osteotomy area, thus reducing the occurrence of adverse events. This topic needs further research.
